# Factors associated with poor prognosis among patients admitted with heart failure in a Nigerian tertiary medical centre: a cross-sectional study

**DOI:** 10.1186/1471-2261-8-16

**Published:** 2008-07-22

**Authors:** Kamilu M Karaye, Mahmoud U Sani

**Affiliations:** 1 Department of Medicine, Bayero University, Kano, Nigeria

## Abstract

**Background:**

Heart failure is a major and growing public health problem worldwide. The prognosis of Heart Failure (HF) is uniformly poor despite advances in treatment. The aims of the present study were to determine the causes of HF among patients admitted to a Nigerian tertiary medical centre, to determine the prevalence of factors known to be associated with poor prognosis among these patients, and to compare the factors and causes between males and females.

**Methods:**

The study was cross-sectional in design, carried out on eligible patients who were consecutively admitted with HF, in Aminu Kano Teaching Hospital, Kano, Nigeria. The following established factors associated with poor prognosis of HF were assessed: low Left Ventricular Ejection Fraction (LVEF) of ≤ 40%, anaemia, renal impairment, cardiac rhythm disturbances on the electrocardiogram, prolonged corrected QT interval (QT_c_), complete Left Bundle Branch Block (LBBB) and advanced age.

**Results:**

A total of 79 patients were studied over a six-month period. Forty four (55.7%) of these patients were males while the remaining 35 (44.3%) were females. The most prevalent prognostic factor was low LVEF found in a total of 35 patients (44.3%), while the least prevalent was complete LBBB found in two male patients only (2.53%). The commonest cause of heart failure in all patients and males was hypertensive heart disease, found in a total of 45 patients (57.0%), comprising of 33 male (73.3%) and 12 female patients (26.7%) (*p *= 0.0003). Cardiomyopathies were the commonest causes in females, the predominant type being peripartum cardiomyopathy found in 11 (31.4%) female patients. Acute myocardial infarction has emerged to be an important cause of HF in males (13.6%) with a high in-hospital mortality of 66.7%.

**Conclusion:**

The most prevalent factor associated with poor prognosis was low LVEF. Hypertensive heart disease and cardiomyopathies were the most common causes of HF in males and females respectively. The findings of the study should guide decision-making regarding management of HF patients.

## Background

Heart Failure (HF) is a major public health problem worldwide, affecting approximately 5 million Americans and 0.4–2% of the general European population.[1,2] Though there are no large scale studies on HF in Africa including Nigeria, previous studies have revealed a hospital admission rate of 3–7% for HF across the region, which is similar to the rate in developed countries of Western Europe and America.[3] Unfortunately, the prognosis of HF is uniformly poor despite advances in treatment. Half of the patients carrying the diagnosis of HF are likely to die within 4 years, while more than 50% of those with severe HF are likely to die within one year.[2] Women with HF differ from men in their demographic and clinical characteristics, and possibly in their response to HF treatment.[1] Several studies from the western world have shown that women with HF have better prognosis.[4] However, women in developing countries are an economically and socially disadvantaged group, hence the emphasis on them in the Millennium Development Goals of the United Nations.[5]

The aims of the present study were to determine the causes of heart failure among patients admitted to a Nigerian tertiary medical centre, to determine the prevalence of factors known to be associated with poor prognosis among these patients, and to compare the factors and causes between males and females.

## Methods

The study was carried out in Aminu Kano Teaching Hospital, which is affiliated to Bayero University, Kano, Nigeria. It is the only tertiary health centre in the most populous Nigerian State of Kano, North-Central Nigeria. It receives referrals from hospitals in Kano and Jigawa States, as well as from parts of neighbouring States including Katsina, Yobe and Bauchi States.

The Research Ethics Committee of Aminu Kano Teaching Hospital reviewed and approved the study protocol. All recruited patients gave written informed consent to participate in the study. The study conformed to the principles outlined in the Declaration of Helsinki, on the ethical principles for medical research involving human subjects.[6]

The study was cross-sectional in design. Eligible patients were recruited consecutively into the study after obtaining informed consent. For patients to be eligible, they had to meet the following criteria: age of at least 15 years or older, confirmed diagnosis of HF, and admission to the medical wards of the study centre. All other patients were excluded from the study. Minimum sample size was 68 patients calculated with a validated formula [7], applying a precision of 10% and prevalence of HF of 10%. This prevalence of HF was estimated from the admission records of previous 6 months of the medical wards. All recruited patients were either evaluated immediately after admission or within the first 48 hours. Detailed history was taken from all patients and comprehensive physical examination carried out. The patients were investigated according to the recommendations of standard guidelines for the management of patients with HF [1,2]. Other investigations (e.g. blood cultures) were also carried out when indicated. Echocardiography was performed by the authors, according to the recommendations of the American Society of Echocardiography.[8]

Heart failure was defined according to the recommendations of the European Society of Cardiology.[2] The following established prognostic factors for heart failure were assessed:[1,2] Left Ventricular Ejection Fraction (LVEF) of ≤ 40%, anaemia, renal impairment, cardiac rhythm disturbances, prolonged corrected QT interval (QT_c_), complete Left Bundle Branch Block (LBBB) and advanced age.

Anaemia was defined as Packed Cell Volume of <39% and <36% in men and women respectively. Renal impairment was defined as serum creatinine of >176 μmol/l (>2 mg/dl). Prolonged QT_c _on Electrocardiogram (ECG) was defined as a value >440 ms and >460 ms in males and females respectively, or more than 500 ms if there was ventricular depolarization abnormality.[9] Cardiac rhythm disturbances and complete LBBB were also defined according standard guidelines.[9] Advanced age was defined as age of ≥ 65 years.

In patients with systemic hypertension, the diagnosis of hypertensive heart disease was based on the presence of any abnormality that is causally related to hypertension and without alternative explanation, on the ECG and echocardiogram. These abnormalities include concentric or eccentric Left Ventricular Hypertrophy (LVH), increased left ventricular mass index, increased left ventricular or left atrial size and volumes, and diastolic or systolic left ventricular dysfunctions.[10] Ischemic heart disease was diagnosed if the subject had either positive history of typical angina or acute myocardial infarction, and/or typical ECG abnormalities of acute myocardial infarction or myocardial ischemia, plus ventricular regional wall motion abnormality on 2D echocardiography.[11] Wall Motion Score Index was calculated for each patient with regional wall motion abnormality, and their left ventricular systolic and diastolic functions were evaluated.[12,13] Acute myocardial infarction was defined according to the recommendations of the joint European Society of Cardiology/American College of Cardiology Committee.[14] Rheumatic mitral regurgitation and aortic regurgitation were defined by the presence of valvular regurgitation in two planes on Doppler echocardiography and with the following features on 2D echocardiography: thickened and retracted leaflets and subvalvar apparatus, restricted leaflet mobility, and poor coaptation of the leaflets in systole which could be worsened by the dilatation of the valve annulus.[15,16] Rheumatic mitral stenosis was defined by the presence of thickened and/or calcified mitral leaflets and subvalvar apparatus, decreased E-F slope (on M- Mode echocardiography), 'hockey-stick' appearance of the anterior mitral leaflet in diastole, immobility of the posterior mitral leaflet, and narrowed 'fish-mouth' orifice of the mitral valve in the short-axis view measurable with planimetry (with valve area of ≤ 2.0 cm^2^) or Doppler echocardiographic techniques (the diastolic pressure half-time method or the continuity equation).[15,16] Rheumatic aortic stenosis was defined by the presence of thickened or calcified and immobile aortic valve cusps, with commissural fusion causing a narrowed orifice (valve area of ≤ 1.5 cm^2^), and almost invariably occurring with rheumatic mitral valve disease.[15,16] Dilated cardiomyopathy was defined by the presence of dilated left ventricle (with or without dilatation of the other 3 cardiac chambers) with global systolic and diastolic dysfunctions.[17] Peripartum cardiomyopathy was diagnosed if echocardiography had revealed features of dilated cardiomyopathy (mentioned above) in the absence of a demonstrable cause or other structural heart disease, and if disease was identified for the first time within the last trimester of pregnancy or in the first 5 months postpartum.[18]

Data analysis was done using SPSS version 16.0. Means and standard deviations were computed for quantitative variables and the Student's t-test was used to compare means. The Chi-squared or Fisher's exact tests were used to test for significance among categorical variables. A *p*-value of < 0.05 was considered significant.

## Results

A total of 79 patients were admitted to the medical wards of Aminu Kano Teaching Hospital with a clinical diagnosis of HF over a six-month period (May – October 2007), and all of them consented to participate in the study. Forty four (55.7%) of these patients were males while the remaining 35 (44.3%) were females, giving a male:female ratio of 1.26:1. The mean age of all the patients was 46.90 ± 17.89 years. Though males had higher mean age (49.09 ± 16.39 years) compared to females (44.14 ± 19.53 years), the difference was not statistically significant (*p *= 0.224).

Table 1 shows factors associated with poor prognosis among the studied patients and compares males with females. All the studied patients had at least one such prognostic factor. The most prevalent prognostic factor was LVEF ≤ 40 found in 44.3% of all patients, while the least prevalent was complete LBBB found in two male patients only (2.5%). Prolonged QT_c _was significantly more prevalent in males (*p *= 0.0037). Atrial Fibrillation (AF) was the most prevalent rhythm abnormality recorded from 15 patients (19.0%), comprising of 8 male (18.2%) and 7 female patients (20.0%) (*p *= 0.838). Premature ventricular complexes were recorded from 7 patients (8.9%), comprising of 3 male and 4 female patients (*p *= 0.474). Furthermore, atrial flutter and complete Right Bundle Branch Block (RBBB) were absent among females but recorded from 2 (4.6%) and 7 (15.9%) male patients respectively.

**Table 1:** Prognostic factors for heart failure among studied subjects

**Factors**	**All patients **	**Males**	**Females **	***p*-value**
	**N = 79**	** N = 44**	**N = 35**	
**LVEF ≤ 40%**	35(44.3%)	20(45.5%)	15(42.9%)	0.817
**Anaemia**	32(40.5%)	21(47.7%)	11(31.4%)	0.143
**Abnormal rhythm**	24(30.4%)	13(29.6%)	11(31.4%)	0.857
**Prolonged QT_c_**	22(27.9%)	18(40.9%)	4(11.4%)	0.004*
**Advanced age**	16(20.3%)	7(15.9%)	9(25.7%)	0.399
**Renal impairment**	9(11.4%)	7(15.9%)	2(5.7%)	0.157
**Complete LBBB**	2(2.5%)	2(4.6%)	0(0%)	__

Key: N, Number of patients; LVEF, Left Ventricular Ejection Fraction; LBBB, Left Bundle Branch Block. * *p*-value statistically significant. Values are presented as N with percentages in parenthesis.

Table 2 describes the aetiologies of heart failure, while Table 3 describes distribution of the prognostic factors among patients grouped by heart failure aetiology. These Tables show that the most common aetiology of HF was hypertensive heart disease found in a total of 45 patients (57.0%), comprising of 33 male (73.3%) and 12 female patients (26.7%) (*p *= 0.0003). Sixteen patients (35.6%) with hypertensive heart disease had LVEF ≤ 40%, while 18 of them (40.0%) had anaemia. One-fifth of them (20.0%) were found to have atrial fibrillation and impaired renal function.

**Table 2:** Aetiology of heart failure among studied subjects

**Aetiology**	**All patients **	**Males **	**Females **	***p*-value**
	**N = 79**	**N = 44**	**N = 35**	
**Hypertensive heart disease**	45(57.0%)	33(75.0%)	12(34.3%)	<0.001*
**Peripartum cardiomyopathy**	11(13.9%)	__	11(31.4%)	__
**Rheumatic heart disease**	10(12.7%)	6(13.6%)	4(11.4%)	0.769
**Pericardial disease**	10(12.7%)	5(11.4%)	5(14.2%)	0.698
**Dilated cardiomyopathy**	8(10.1%)	6(13.6%)	2(5.7%)	0.246
**Acute myocardial infarction**	6(7.6%)	6(13.6%)	0(0%)	__
**Cor-pulmonale**	2(2.5%)	0(0%)	2(5.7%)	__

Key: N, Number of patients; * *p*-value statistically significant. Values are presented as N with percentages in parenthesis.

**Table 3:** Distribution of prognostic factors among heart failure patients grouped by aetiology

**Factors**	**Hypertensive heart disease**	**Peripartum cardiomyopathy**	**Dilated cardiomyopathy**	**Rheumatic heart disease**	**Acute myocardial infarction**
	**N = 45**	**N = 11**	**N = 8**	**N = 10**	**N = 6**
**LVEF ≤ 40%**	16(35.6%)	9(81.8%)	6(75.0%)	2(20.0%)	6(100.0%)
**Anaemia**	18(40.0%)	4(36.4%)	4(50.0%)	0	2(33.3%)
**Abnormal rhythm**	11(24.4%)	0	0	6(60.0%)	2(33.3%)
**Prolonged QT_c_**	11(24.4%)	2(18.2%)	4(50.0%)	0	2(33.3%)
**Advanced age**	9(20.0%)	0	0	0	2(33.3%)
**Renal impairment**	9(20.0%)	0	0	0	0
**Complete LBBB**	2(8.9%)	0	0	0	0

Key: N, Number of patients; LVEF, Left Ventricular Ejection Fraction; LBBB, Left Bundle Branch Block. * *p*-value statistically significant. Values are presented as N with percentages in parenthesis.

Cardiomyopathies were the commonest causes of heart failure among females, affecting 13 of them (37.1%). On its own, peripartum cardiomyopathy was the 2^nd ^most common cause of heart failure among all female patients (31.4%) as well as among all patients (13.9%) in the series (see Table 2). Patients with peripartum cardiomyopathy were significantly younger than other female patients with heart failure; mean age of 26.4 ± 5.7 Vs 52.3 ± 18.1 years (*p *< 0.001). They also had lower parity compared to the other female patients; 3.2 ± 3.1 Vs 5.5 ± 4.0 (*p *= 0.116). In addition, 9 of them (81.8%) had LVEF ≤ 40% while 4 of them (36.4%) had anaemia. The mean systolic and diastolic blood pressures of these patients were 106.4 ± 13.6 and 70.0 ± 13.4 mmHg respectively. None of these patients was known to be hypertensive. Furthermore, six of the patients with peripartum cardiomyopathy (54.6%) had LV thrombus, and 4 of these patients developed cardioembolic stroke events.

Rheumatic heart disease was found in a total of 10 patients (12.7%), six of whom (60.0%) had multi-valvular disease. The most frequent valvular lesion was rheumatic mitral regurgitation affecting 8 patients (10.1%) comprising of 2 male and 6 female patients (*p *= 0.065). In the patients with rheumatic heart disease, 20.0% had LVEF ≤ 40%, 40% had atrial fibrillation while another 20% had atrial flutter.

Pericardial disease was the cause of heart failure in 10 patients (12.7%). Two of these patients (10.0%) had constrictive pericarditis, while the rest had either effusive pericarditis or effusive myopericarditis. Four of the patients (40.0%) had both tuberculosis and acquired immunodeficiency syndrome, while five of them (50.0%) gave history of systemic hypertension. Five of these patients (50.0%) had advanced age, 40% had anaemia, and 30% had prolonged QT_c _while 20% had rhythm disorders.

Acute myocardial infarction was the cause of heart failure in 6 male patients (13.6% of all males). All of them had LVEF ≤ 40% and were hypertensive. Anaemia, rhythm disorders and prolonged QT_c _were found in one-third of them (see Table 3).

Eight of the studied patients died during the hospitalisation giving an in-hospital mortality rate of 10.1%. All the patients who died had LVEF ≤ 40. There was tendency of higher hospital mortality among males (6 patients, 13.6%) compared to females (2 patients, 5.7%) but this was not statistically significant (p = 0.246). The mortality was highest among patients with acute Myocardial Infarction (4 patients; 66.7%), followed by patients with rheumatic heart disease (2 patients; 20.0%) and hypertensive heart disease (2 patients; 4.4%).

## Discussion

This study has assessed the causes of HF among patients consecutively admitted with HF to a Nigerian tertiary medical centre. It has also assessed the prevalence of factors known to be associated with poor prognosis, and has compared the factors and aetiologies between males and females.

The patients in this study were relatively young with a mean age of 46.90 ± 17.89 years. This finding is similar to that of a study carried out in Accra (Ghana) on patients consecutively admitted with HF (mean age of 42.3 ± 0.9 years).[19] Our finding is in contrast to what was reported from the United States of America (USA) (61 ± 18 years) and Europe (71.3 ± 12.7 years) on the mean ages of patients hospitalised for HF.[20,21] It is known that in non-Western countries, cardiovascular diseases including HF tend to occur a decade or two earlier than they do in Western countries; nearly half occur before 70 years of age, whereas only one fifth occur so early in the West – a difference attributable to both the earlier occurrence of cardiovascular events and the lower level of clinical care available.[22]

In the present study, the prevalence rates of several of factors associated with poor prognosis were high with few gender differences, mainly in the prevalence of complete LBBB recorded from 2 male patients and prolonged QT_c _(higher in males) (*p *= 0.004). The finding of Complete LBBB and RBBB exclusively among male patients might have contributed to their higher prevalence of prolonged QT_c_. Moreover, hypertensive heart disease was more prevalent among males (*p *= 0.0003). This disorder was found to be associated with high prevalence of LVH in Kano,[23] and cardiac hypertrophy is a factor that independently prolongs QT_c_.[24]

Low LVEF (≤ 40%) was recorded from 44.3% of all patients in our series. The prevalence was slightly higher in males (45.5%) compared to females (42.9%), but the difference was not statistically significant (*p *= 0.668). It is possible that a larger sample size might demonstrate a significant statistical difference between the sexes. In fact, a large study carried out across Europe on patients hospitalized with HF had shown a significantly higher prevalence rate of low LVEF (≤ 40%) in males (47%) compared to females (24%) (*p *< 0.001).[21] This appears to be the general trend in developed countries.[4] Gender-related differences in geometric remodelling and earlier onset of impaired LV systolic function in males have been described in animal models, and in humans with aortic valve stenosis and systemic hypertension.[25]

In the present study, anaemia was found in a total of 40.5% of all patients, and in almost half of male (47.7%) and one-third of female (31.4%) patients respectively (*p *= 0.143). This finding is similar to what Olubodun previously reported (36.7%) from South-Western Nigeria among patients with hypertensive heart failure.[26] Our finding was however higher than that of Oyoo and Ogola (13.2%) reporting from Nairobi (Kenya),[27] but lower than that of Howie-Esquivel and Dracup (50%) reporting from Northern California (USA) [20]. Several factors are known to be associated with low haematoctrit and anaemia among HF patients, including sex and age (older women), race (Caucasians), preserved LV systolic function and high serum creatinine.[28]

Atrial Fibrillation (AF) was the most prevalent rhythm abnormality recorded from 15 patients (19.0%), comprising of 8 (53.3%) male and 7 female (20%) patients (*p *= 0.838). The prevalence of AF is similar to that reported by Familoni *et al *(20.7%) among HF patients in Shagamu (South-Western Nigeria).[29] There are conflicting data from the industrialized countries regarding gender preponderance of AF; some studies showing higher prevalence of AF among males with HF [4], while others show female preponderance [21].

The commonest aetiology of HF among all patients and both sexes in our series was hypertensive heart disease. This finding is similar to that of other studies in Nigeria.[29,30] In comparison, the prevalence of hypertensive heart disease was significantly higher in males with HF compared to females (*p *< 0.0001). This is probably a reflection of the higher prevalence of systemic hypertension (defined using systolic/diastolic blood pressure cut-offs of ≥ 160/90 mmHg respectively), among males (23.8%) compared to females (17.7%) in the general population of Kano, according to the 1997 report of Nigerian Non-Communicable Diseases Committee.[31] The present study has described the profile of prognostic factors for HF secondary to hypertensive heart disease. All the studied prognostic factors were found in the patients with this disease, and six out of the seven factors was found in at least 20% of the patients. History of hypertension and its cardiac complications were also detected in some patients with other aetiologies including acute myocardial infarction and pericardial disease. Hypertensive heart disease was also one of the three causes of in-hospital mortality, responsible for 4.4% of all HF deaths. This degree of morbidity and mortality is not surprising given that a previous study in the same study centre on patients with systemic hypertension, had shown that majority of untreated (68.5%) and treated (75.6%) hypertensives were at very high risk for cardiovascular events, and that blood pressure control was achieved in only 14.3% of those on treatment.[23] The preventive approach to this problem should therefore aim at early identification and treatment of hypertension and other cardiovascular risk factors, and improving blood pressure control.

Cardiomyopathies were the commonest aetiology of heart failure among females in our series. Peripartum cardiomyopathy was the aetiology of HF in almost one-third (31.4%) of all female patients. This disorder has been recognized as an important cause of HF in Kano and other northern Nigerian cities such as Katsina, Zaria and Sokoto, for more than 30 years.[30,32-34] In the present study, patients with peripartum cardiomyopathy were found to be younger (26.4 ± 5.7 Vs 52.3 ± 18.1 years) and had lower parity (3.2 ± 3.1 Vs 5.5 ± 4.0), compared with the other female patients. About 82% of them had LVEF ≤ 40% while 36% of them had anaemia. Thrombus in the LV was found in 60% of the patients and 36.4% of them suffered stroke. In addition, none of them was found to be hypertensive or gave a history of such during previous pregnancies or puerperal periods. The behaviour of their blood pressure on long-term follow up is our future research interest. Peripartum cardiomyopathy has been extensively studied in Zaria, a city 120 kilometres from Kano. Though the language and cultures of the inhabitants of the two cities are similar, the Zaria patients differ from those in Kano in several respects. In Zaria, the risk for peripartum cardiomyopathy was increased by older age and higher parity, and 22% of them developed hypertension during follow up for 2–5 years.[35] Peripartum cardiomyopathy has also been studied in Sokoto, a city about 500 kilometres from Kano, and with similar language and cultural practices.[34] The disorder was also the commonest cause of consecutive admissions for heart failure among females, responsible for 60% of the cases. About one-third of these Sokoto patients were primiparous, 12.3% of them had intracardiac thrombus and 3.1% had atrial fibrillation. However, transient hypertension occurred in 27.7% of them, contrary to our finding but in support of the Zaria experience. Several of our findings are in agreement with those of Sliwa *et al *[36] and Fett *et al *[37] who reported from South Africa and Haiti respectively. The studied patients in both studies were all of African descent. In these studies, the average parity was 3–4 and at least 20% of the patients were primiparous. History of hypertension was obtained from only 2 patients (2%) and 4 patients (4%) among those studied in South Africa and Haiti respectively. The patients were however older than our patients. The mean age of the South African patients was 31.6 ± 6.6 years and 31.8 ± 8.1 years for the Haitians. From the foregoing, it is clear that peripartum cardiomyopathy is an important cause of morbidity and mortality among the indigenous populations of Kano and other parts of North-Central Nigeria, as well as in some parts of the developing world. It can also be said that peripartum cardiomyopathy is a heterogeneous disease. Unfortunately, the aetiology is still unknown but likely to be multifactorial. Research in this area is hampered by its rarity in the developed world where funds and support are concentrated.

About 30 years ago, acute myocardial infarction was reported to be non-existent among Northern Nigerians.[33] The pattern has however changed over the years, across the country. In the present study, acute myocardial infarction was the aetiology of HF among 7.6% of all patients, found exclusively among males. A recent study in South-Western Nigeria among patients admitted with HF has similarly revealed a new trend; ischemic heart disease was the aetiology in 8.5% of patients.[29] In our series, all the affected patients had low LVEF, and at least one-third of them had advanced age, anaemia, abnormal rhythm, and prolonged QT_c_. Unfortunately, facilities for coronary artery angiography, interventions and surgery were not available at the study centre or in the North-Central part of the country. The interplay of these several factors was responsible for the high mortality suffered by the affected patients (66.7%). This in-hospital mortality is higher than what is obtained in countries with developed health care systems (15–20%).[38]

Other important causes of HF include rheumatic heart disease, pericardial disease and cor pulmonale, in agreement with earlier studies in Nigeria and other African nations.[19,27,29,30]

Eight patients (10.1%) died while on admission comprising of 6 male (13.6%) and 2 female (5.7%) patients, without statistically significant gender difference (*p *= 0.246). However, our finding seems to support the widely reported higher mortality in men with HF compared to women.[4] A common characteristic of these patients was low LVEF. It is known that LV systolic dysfunction as assessed by LVEF is the single most important predictor of outcome among patients with symptomatic heart muscle disease.[39] As Sub-Saharan Africa (SSA) is believed to be undergoing rapid epidemiologic transition, it is expected that cardiovascular diseases including HF and acute myocardial infarction, will soon become even more important as causes of morbidity and mortality.[3] Unfortunately, most of SSA is still not yet ready to tackle the burden of such diseases, at the risk of serious consequences.

Though the minimum sample size requirement was met, the most important limitation of this study has become the sample size. A larger sample size that would describe a robust prognostic factor profile of the HF patients, as well as possible significant differences between the groups is desirable. Unfortunately, the achievement of this desire is hampered by several factors including lack of adequate manpower, lack of financial support and frequent industrial actions by various professional groups. It is common knowledge that the costs of medical services in most developing countries including Nigeria are fully shouldered by the patients themselves. Sadly, some patients cannot even afford to pay for the admission or for life-saving drugs such as frusemide. Other important limitations include lack of facilities for diagnosis including coronary artery angiography, as mentioned earlier. Despite the limitations, the results of this study would contribute towards bridging the gap of knowledge on HF in the developing world, and generate hypotheses for new researches. Fortunately, a large multicentre study on HF is being planned in Nigeria. It is hoped that this will minimise the problems of limited manpower and resources encountered at single centres.

## Conclusion

This study has revealed that subjects admitted with HF in the study centre were relatively young and had several factors associated with poor prognosis The most prevalent were anaemia, low LVEF and rhythm disorders; each of them affected >30% of all patients. The commonest aetiology of HF among all patients and males in our series was hypertensive heart disease. Peripartum and dilated cardiomyopathies were the commonest causes of HF among females. Acute myocardial infarction has emerged as an important cause of HF among males with relatively high mortality.

Majority of these prognostic factors and aetiologies are correctable or avoidable. It is hoped that these findings would guide appropriate management of such patients, with the hope of minimizing the mortality rate.

## Competing interests

The authors declare that they have no competing interests.

## Authors' contributions

KMK conceptualized and designed the study, acquired, analyzed and interpreted data, ensured data quality, carried out echocardiography, drafted the manuscript and gave approval to its final version. MUS acquired data, carried out echocardiography, critically revised the manuscript and gave approval to its final version.

## Pre-publication history

The pre-publication history for this paper can be accessed here:


